# Autoimmune Hepatitis (AIH) in Acquired Immune Deficiency Syndrome (AIDS): A Case Report and Review of Literature

**DOI:** 10.1155/2019/5326428

**Published:** 2019-06-09

**Authors:** Jordan Roussel, Sudha Pandit, Paul Jordan, Moheb Boktor, Kurt Knowles, Nester Dela Cruz, Hrishikesh Samant

**Affiliations:** ^1^Department of Internal Medicine, Louisiana State University Health Sciences Center, Shreveport, LA, USA; ^2^Department of Gastroenterology and Hepatology, Louisiana State University Health Sciences Center, Shreveport, LA, USA; ^3^Department of Pathology, Louisiana State University Health Sciences Center, Shreveport, LA, USA

## Abstract

The common causes of abnormal liver chemistries in human immunodeficiency virus (HIV) infected patients are multifactorial. Diagnosis of autoimmune hepatitis (AIH) in HIV infected patients is intriguing but data is scarce. Unmasking of AIH during immune reconstitution in HIV patients after starting antiretroviral therapy is reported but not with advanced acquired immunodeficiency syndrome (AIDS). Here we present a fascinating case of 32-year-old African-American man with advanced AIDS who presented with elevated transaminases. He was diagnosed with AIH before starting antiretroviral therapy and successfully treated with prednisolone and azathioprine with antiretroviral therapy despite very low CD4 count.

## 1. Introduction

Patients with human immunodeficiency virus (HIV) infection have a wide variety of causes for elevated liver chemistries. Some common etiologies are coinfections such as hepatitis B (HBV), Hepatitis C (HCV), opportunistic infection, and alcoholic and nonalcoholic fatty liver disease, and less common causes include cholecystitis, AIDS related cholangiopathy, and antiretroviral drug toxicity. Autoimmune hepatitis (AIH) is rarely reported as cause of liver function derangement in HIV patient. The relation between HIV and AIH is interesting. The pathophysiology of AIH begins with loss of self-tolerance of T-lymphocytes causing an overwhelming immune cascade leading to autoantibodies to liver cells [1]. On the other hand, HIV is a disease that causes decreased activity of the immune system by infecting and destroying T-lymphocytes and also sometimes developing various antibodies by polyclonal stimulation of B cells. The diagnosis of AIDS is made when a patient with an HIV infection becomes significantly immunocompromised with a severely reduced CD4 count. This very low CD4 cells and severely immunocompromised state is the basis of why developing AIH in a patient with AIDS may be extremely rare. AIH with advanced AIDS, to our knowledge, has never been reported. Furthermore, treating AIH with high dose immunosuppressive medication is usually risky in AIDS patient. We herein report the case of advanced AIDS patient who was diagnosed with AIH before starting antiretroviral therapy and successfully achieved remission of transaminitis on standard immunosuppressive therapy for AIH and antiretroviral therapy without any side effects.

## 2. Case Presentation

**Figure 1:**
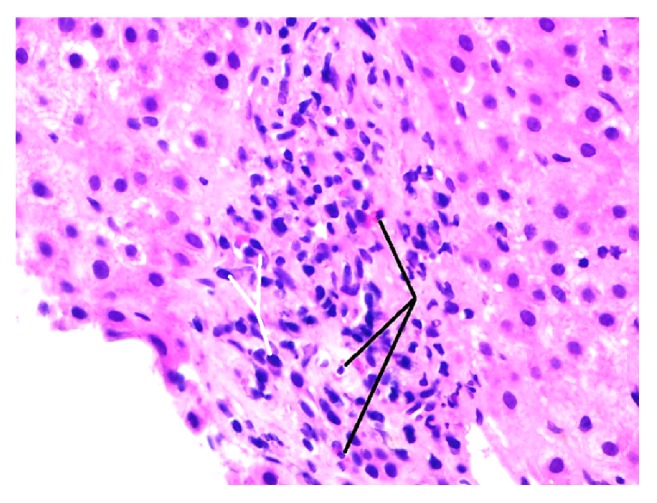
Liver biopsy. The portal triad demonstrates plasma cells (white arrows) and eosinophils (black arrows) consistent with autoimmune hepatitis. H&E stain, high power (400X).

Our patient is a 32-year-old African-American male with past medical history of HIV/AIDS, hypertension, and focal segmental glomerulosclerosis (FSGS) related chronic kidney disease stage 3, who presented to our institution complaining of a two-week history of generalized abdominal pain, associated with jaundice, pruritus, nausea, and vomiting. The patient's HIV/AIDS status was significant for a very low CD4 count of 11 cells/mm3 with a viral load of 64000 IU/ml. Patient had history of nonadherence to antiretroviral therapy and was not taking his medications since his diagnosis of HIV. Review of systems was otherwise normal. Travel history or use of nonprescription medications was negative. Family history was noncontributory. Surgical history included a knee surgery. He had 20-pack year history of cigarette smoking but denied alcohol, illicit drug use, or hepatotoxic medications. Pertinent positives in the physical examination included icteric sclera, hepatomegaly 2 cm below costal margin, and jaundiced skin. Admission labs revealed abnormal liver chemistries: aspartate transferase (AST) 255 U/L, alanine transferase (ALT) 461 U/L, alkaline phosphatase 123 U/L, gamma glutamyl transferase 34 U/L, and total bilirubin 17.4 mg/dL. Viral hepatitis workup was negative which included IgM/Total hepatitis A, IgM hepatitis E, anti-HCV antibody, HCV RNA, HEV RNA, hepatitis B surface antigen, IgM/total hepatitis B core antibody, and HBV DNA viral load. Other infectious disease workups including cytomegalovirus (CMV), Herpes Simplex (HSV), Epstein Barr (EBV), syphilis, and varicella were all negative. Ultrasound liver revealed hepatomegaly. MRCP done for borderline elevated alkaline phosphatase and pruritis but was negative for any biliary pathology. Urine drug screen was negative. Amongst autoimmune markers, anti-nuclear antibody (ANA), and liver kidney microsomal (LKM)-1, anti-mitochondrial antibody (AMA) was negative, but anti-smooth muscle antibody (SMA) titers by ELISA came positive at 1:84 with elevated immunoglobulin G levels of 3540 g/L (twice upper limit of normal levels). Further evaluation with core liver biopsy revealed chronic lymphoplasmacytic inflammation with abundant plasma cells and minimal interface activity without bile duct damage, necrosis, or fibrosis suggestive of autoimmune hepatitis (Figure 1). Patient fulfilled the Revised Original Score for Autoimmune Hepatitis with score of 20 giving diagnosis as “Definite for AIH” [16, 17]. The patient was started on standard dose of highly active antiretroviral treatment (HAART) and prednisone 30 mg oral daily with close monitoring of liver enzymes. After starting prednisone, the patient improved symptomatically with improvement in liver functions and immunoglobulin levels. Patients CD4 count remained < 50 during the period of liver function improvement. Azathioprine was introduced gradually at 1-1.5 mg/kg on week 2 after confirming normal thiopurine methyltransferase (TPMT) phenotype and subsequently steroid was tapered off successfully. Patient's liver enzymes normalized completely after 9 months with continued azathioprine and HAART.

## 3. Discussion

Autoimmune hepatitis is a rare cause of transaminitis in HIV infected patients with the more common causes being a coinfection such as HBV and HCV due to the same route of transmission as HIV. Also, opportunistic infection, steatohepatitis, and medications toxicity may coexist causing abnormal liver enzymes in HIV population [18]. In recent study by Virot and collogues investigating the prevalence of autoimmune diseases in HIV patients, only one case of AIH was identified in a cohort of 5186 patients [19].

The HIV virus causes the destruction of the host's T-lymphocytes resulting in an immunocompromised state. Once the CD4 T-lymphocytes are less than 200 or the patient develops an AIDS-defining condition, the term acquired immunodeficiency syndrome (AIDS) can be used. Once the CD4 count drops below 50, it is called “advanced AIDS”. Since AIH results from overactivation of T-lymphocytes, whereas AIDS is a result of depletion of T-lymphocytes, these contrasting processes may be the reason for the low prevalence of AIH in HIV patients. Although it is uncommon, autoimmune disorders like immune thrombocytopenic purpura, antiphospholipid syndrome, sarcoidosis, SLE, and Graves' disease have been described in HIV [20]. Pathogenesis of AIDS is known to initiate autoimmune phenomenon by polyclonal stimulation of B cells by HIV virus itself and development of hypergammaglobulinemia in proinflammatory milieu. In HIV, immune system is known to attack itself and form circulating autoimmune antibodies [21]. Vergani and collogues showed cross-reaction between anti-liver kidney microsome (LKM)-1 antibodies (involved in type 2 AIH) against CYP2D6 and HCV, HSV, and CMV [22]. Nowadays more cases of autoimmune diseases in HIV are reported with increasing use of HAART for treatment of HIV, suggesting unmasking of an underlying autoimmune dysfunction with immune reconstitution which predominantly involve CD8 cells [2, 4]. However, in the present case, at the time of diagnosis of AIH, patient was not taking HAART and had very low CD4 count without any evidence of coinfection.

We searched PubMed database for relevant English literature with the search terms of “Autoimmune hepatitis” and “human immunodeficiency virus”, “Acquired Immunodeficiency syndrome” and identified 14 case reports/series of 35 patients with the concomitant diagnosis of AIH and HIV only without any coinfection (Table 1). In these previous reported cases/series, the range of CD4 count at the time of AIH diagnosis was between 174 and 1011 cells/mm3 with a viral load between undetectable and 27,000 IU/ml [2–15]. Our patient's CD4 count was just 11 cells/mm3 and viral load was 64,000 IU/ml at the time of AIH diagnosis. Also, cases described in the literature thus far only had HIV infection with AIH diagnosis, but our patient is unique in the sense that he had advanced AIDS when he was diagnosed with AIH.

Although there are more common causes of transaminitis in HIV/AIDS patients, an autoimmune hepatitis can still be a possibility. Physicians must be aware of this completely treatable but challenging “AIH in HIV/AIDS” diagnosis which frequently will need detailed biochemical, viral, autoimmune workup, and likely liver biopsy. Furthermore, there are no guidelines in the treatment of AIH in HIV patients. Standard immunosuppressive therapy, such as steroids and azathioprine, is usually thought to be high-risk in already immunocompromised HIV patients. There are only two reported cases, where patient's condition improved with HAART alone [3, 7]. Prior retrospective case reports showed that standard AIH therapy was successful in well controlled HIV on HAART (CD4 >250 and undetectable HIV viral load). In our case, despite very low CD4 count and high viral load, the patient showed complete remission of liver functions following the administration of prednisolone and azathioprine for AIH and starting HAART for AIDS.

## 4. Conclusion

Our case highlights that etiological workup for elevated liver chemistry in HIV/AIDS should include autoimmune hepatitis, irrespective of degree of immunosuppression and despite severe immunocompromised state, standard treatment for AIH can be used safely with HAART under close supervision.

## Figures and Tables

**Table 1:** Review of reported cases/case series of HIV with AIH.

Author	Case	Age	Sex	CD4 at diagnosis (cells/mm3)	Viral Load diagnosis (IU/ml)	AIH score	ART	AIH Treatment	Outcome
Daas et al. [2]	1	42	F	232	Undetectable	Definite	Yes	Steroids	Remission

Tan-Tam et al [3]	1	59	M	>150	-	-	Yes	Transplant	Transplant

	3	29	M	174	27,732	Probable	Yes	None	Remission
Puius et al. [4]		45	F	297	Undetectable	Probable	Yes	Steroids +AZT	Remission
		65	F	922	Undetectable	Definite	Yes	Steroids	Remission

Wan et al. [5]	2	54	M	357	5,104	Probable	Yes	Steroids	Remission
		49	F	286	69,318	Definite	Yes	Steroids	Remission

Kaku et al. [6]	1	43	M	638	13,000	Probable	Yes	Steroids	Remission

O'Leary et al. [7]	1	44	F	526	Undetectable	Definite	Yes^1^	Steroids +AZT	Remission

German et al. [8]	1	38	M	216	Undetectable	Probable	Yes	None	Remission

Parekh et al. [9]	2	21	M	753	7,900	Probable	No	Steroids +AZT	Remission
		59	F	483	Undetectable	Definite	Yes	Steroids +AZT	Remission

Hagel et al [10]	1	52	M	641	Undetectable	Probable	Yes	Steroids	Remission

	5	33 to 60	M	1011	Undetectable	-	Yes	Steroids +AZT	Remission
			F	456	Undetectable		Yes		
Kia et al. [11]			F	783	Undetectable		Yes		
			F	688	Undetectable		Yes		
			F	653	Undetectable		Yes		

Ofori et al. [12]	2	40	M	832	Undetectable	Probable	Yes	Steroids	Remission
		44	F	823	Undetectable	Probable	Yes	Steroids	Remission

Murunga [13]	9	23 to 45	1 M 8 F	253-876	Undetectable	-	Yes	Steroids	Remission

Iordche [14]	4	-	-	488.4	Undetectable	-	Yes	Steroids +AZT	Remission

Zoboli [15]	2	38	M	762	8072	Definite	Yes	Steroids +AZT	Remission
		70	F	938	<50	Definite	Yes	Steroids	Remission

Our patient	1	32	M	11	64,000	Definite	No	Steroids +AZT	Remission

AZT: azathioprine, AIH: autoimmune hepatitis, and ART: antiretroviral therapy.

## Data Availability

Data sharing is not applicable to this article as no datasets were generated or analyzed during the current study.
